# Levels of depressive symptoms in cardiac patients attending cardiac rehabilitation with a history of depression: pre Covid-19 and Covid-19 period comparison

**DOI:** 10.1186/s12872-022-02867-4

**Published:** 2022-09-28

**Authors:** Serdar Sever, Alexander Stephen Harrison, Patrick Doherty

**Affiliations:** 1 Department of Health Sciences, Faculty of Science, University of York, ATB/255 Seebohm Rowntree Building, York, UK; 2 Faculty of Health Sciences, Usak University, Uşak, Türkiye

**Keywords:** COVID-19, Cardiovascular disease, Cardiac rehabilitation, Acute depressive symptoms, History of depression, Observational study

## Abstract

**Background:**

The large-scale changes in cardiac rehabilitation (CR) programme delivery in response to COVID-19 has led to diminished provision. The influence of these service changes on the depression symptoms of patients in CR programmes is unknown. Our study investigated the extent of depressive symptoms prior to and during the COVID-19 periods in patients with a previous history of depression at the start of CR.

**Methods:**

Use of Registry routine practice data, National Audit of Cardiac Rehabilitation (NACR), from COVID-19 period Feb 2020 and Jan 2021, as well as pre COVID-19 period Feb 2019 and Jan 2020, was extracted. Depressive symptoms were defined according to Hospital Anxiety and Depression Score ≥ 8. Chi-square tests and independent samples t-tests were used to investigate baseline characteristics. Additionally, a binary logistic regression to examine the factors associated with high levels of depressive symptoms.

**Results:**

In total 3661 patients with a history of depression were included in the analysis. Patients attending CR during COVID-19 were found to be 11% more likely to have high levels of acute depressive symptoms compared to patients attending CR prior to COVID-19. Physical inactivity, increased anxiety, a higher total number of comorbidities, increased weight, and living in the most deprived areas were statistically significant factors associated with high levels of acute depressive symptoms at the start of CR following multivariate adjustments.

**Conclusion:**

Our research suggests that following a cardiac event patients with prior history of depression have high levels of acute depressive symptoms at CR baseline assessment. This finding exists in both the pre Covid-19 and Covid-19 periods in patients with a history of depression.

## Background

Depression is associated with increased mortality and cardiac morbidity in cardiovascular disease (CVD) patients which is well evidenced in previous studies [1, 2] and recommended to be accepted as a risk factor for mortality and worse cardiac outcomes [3]. Depression is common in cardiac patients, with a prevalence of 20% [4], and linked to increased health care costs [5]. CR is a multi-component programme that aims for the comprehensive management of CR patients and provides secondary prevention [6]. Recent clinical guidelines view depression as a risk factor associated with poor cardiac prognosis and outcomes and these authors recommend assessment and management of depression as part of core CR [3, 6–8].

On 11 March 2020, a pandemic was declared by the World Health Organisation due to coronavirus disease (COVID-19) [9]. COVID-19 creates multiple challenges for public health and medical communities due to the rapid increase in the number of cases of this infectious disease caused by severe acute respiratory syndrome [10]. Several serious public health measures were embraced by local governments to reduce the impact of the disease and the risks; for example, work-from-home arrangements, the shutdown of non-essential services, school suspensions, and quarantines for people who are coming back from overseas.

Depression is sensitive to traumatic events and preceding studies, investigating the impact of epidemics or disasters, have shown that traumatic events are associated with increased depression levels in the populations that are affected by the events [11]. SARS outbreak and Ebola virus were other epidemics after which an increase in depressive symptoms was recorded [12, 13]. Preliminary findings of recently published studies have shown the association of COVID-19 with depression in health care workers [14], and in the general population [15, 16]. However, there is scarce evidence investigating the impact of attending CR during the COVID-19 period on the levels of depressive symptoms in patients with cardiovascular disease. To the best of our knowledge, no previous studies have investigated levels of depressive symptoms by applying HADS measurements in patients with a history of depression at the commencement of CR during the COVID-19 pandemic. Although a recent study has employed HADS to investigate new-onset depressive symptoms by excluding patients with prior history of depression in the cardiac population [17], this study was not inclusive of COVID-19 data, and also examining the patients with a history of depression remains relevant. Furthermore, the assessment of patients' depressive symptoms is still not at the desired levels [18], so more research is needed to expand on specific patient populations as the current study aims to do.

The current research aimed to investigate the percentage of depressive symptoms in UK CR patients during the COVID-19 pandemic and examined the factors associated with high levels of acute depressive symptoms based on HADS score (HADS ≥ 8) before and during COVID-19 in patients with a prior history of depression. This study also investigated the impact of variables that are potentially influential factors such as age, the total number of comorbidities, HADS anxiety, gender, comorbidity anxiety, physical activity, smoking, and Index of Multiple Deprivation (IMD), marital status, and attending CR during the COVID-19 period. Knowing the potential factors linked with a high risk of having acute depressive symptoms may enable CR providers to tailor the intervention to the specific needs of the patients with a history of depression.

## Methods

To report the current study, the strengthening of the reporting of observational studies in epidemiology (STROBE) checklist was utilized [19].

### Data collection

The study population and data were from the National Audit of Cardiac Rehabilitation (NACR). Patient-level data were extracted and analysed. Using routinely collected NACR data an observational study was conducted. The NACR aims to monitor UK CR programmes and thereby advance the quality of service delivery and outcome in CR centres. Individual patient data are collected under section 251 of the NHS Act 2006 and are entered by clinicians onto the NHS Digital data storage system. Due to the exemption, it is not required for the audit to collect consent to be included in the audit. In addition, it is within the 251 exemption and purpose of the audit that research which is performed for the purpose of service improvement is permitted. Due to these factors, ethical approval was not required for the performance and submission of this study. The CR program staff distributes the questionnaires in their services to the patients, receives the responses, and enters the data into the database. NHS Digital has approval that enables collecting patient identifiable data that is anonymised afterward to be made available for NACR; as such it was not necessary to gain individual consent from each patient. Due to the use of anonymised patient data and the data governance agreements between NHS Digital and NACR, there was no need for separate ethical approval. The total number of CR programmes available for NACR in the UK is 233 including centres from England, Northern Ireland, and Whales, and 83% of them, 194 programmes have electronic NACR registration for data entry which enables greater audit coverage [20]. Patient demographics, treatment, medication, and risk factors were included in the data for those who attend CR in the UK. A detailed description of NACR can be found in the recent NACR report [20].

### Participants

**Fig. 1:**
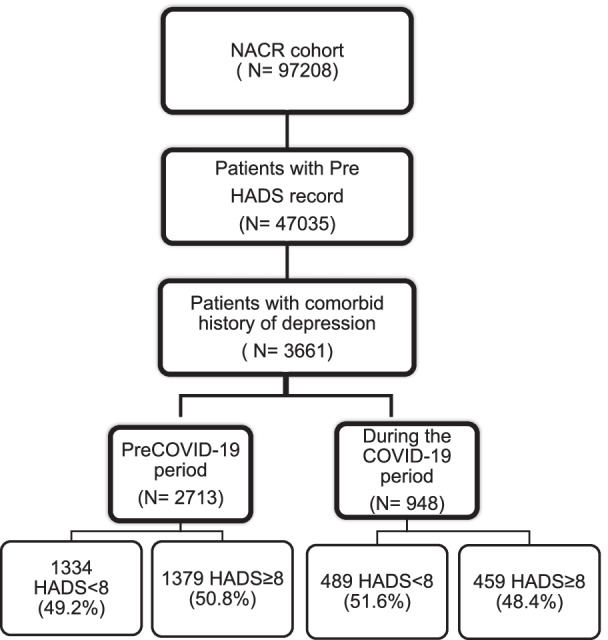
Flow diagram for study sampling

NACR data was used for the analysis and the data was extracted from the NACR between 01 February 2019 and 31 January 2020 for the preCOVID-19 period and 01 February 2020 to 31 January 2021 during the COVID-19 period. The patients with myocardial infarction (MI), heart failure (HF), and who receive the percutaneous coronary intervention (PCI) and coronary artery bypass graft (CABG) treatment were included in the study population due to being recommended in the clinical guidelines [21, 22]. Once the data were extracted between 01 February 2019 and 31 January 2021 including both the preCOVID-19 and during the COVID-19 period, there were a total of 97,208 participants. Afterward, all the eligible patients with baseline HADS measures recorded were selected which was N = 47,035. As the primary aim of the study was to investigate the extent of acute depressive symptoms in patients with a prior history of depression, this specific patient group was selected leading to N = 3661 patients. Of these patients, 2713 patients have attended CR programmes prior to the COVID-19 period and 948 patients during the COVID-19 period as can be seen in Fig. 1. The sample is nationally representative and all attempts to reduce bias were undertaken.

### Measures

Patients with a prior history of depression and valid pre-CR HADS measurements were selected from the NACR data set and an eligible patient population was designated with this approach for the study sample. History of depression is confirmed by CR practitioners with a case note review by looking at the patient’s medical record to confirm pre-diagnosis of depression.

### Hospital Anxiety and Depression Scale

The HADS is used for screening depressive symptoms in clinical practice, and it is an assessment tool employed as a self-answered questionnaire. The HADS is a recommended tool for the assessment of psychosocial health in a CR setting to provide patients with a tailored CR intervention according to patients' needs [6]. There are 14 items included in HADS, 7 items cover anxiety symptoms and 7 depressive symptoms. Each item is assigned with a score of 0–3, based on this minimum of 0 and a maximum of 21 can be received separately for anxiety and depression scores, higher scores relate to worse symptoms. The HADS is recommended to use with cardiac patients as it is found to be a reliable and valid measure for the assessment of anxiety and depression symptoms [23–25]. The clinical cut of point of 8 was set to appoint patients into categories, < 8 indicates patients with low levels of depressive symptoms and ≥ 8 with high levels of depressive symptoms. A systematic review has shown that for the HADS assessment tool, the optimal balance between sensitivity and specificity is constantly achieved at the cut-off score of 8 (sensitivity and specificity for both scales roughly 0.80) for both anxiety and depression [24]. The current study analysis compared patients with HADS < 8 and HADS ≥ 8 in a subgroup of patients with a history of depression and made comparisons between patients attending CR during the COVID-19 period and prior to the COVID-19 period. Additionally, baseline HADS anxiety scores which are routinely reported as parts of HADS were used to see if it was associated with high levels of depressive symptoms at the start of CR.

### Other variables

Age, gender, The English Index of Multiple Deprivation (IMD) [26] and marital status (single/partnered) were patient demographics used in the analysis. IMD was used as a dichotomous variable in the analysis (most deprived quintile/less deprived quintiles), a methodology previously described in detail [27]. Baseline smoking measurements (Pre-CR) are categorised as to whether the patient was a current smoker or non-smoker. Moderate physical activity (150 min. a week), weight (in kilogrammes), and comorbid anxiety were other variables included in the study. The total number of comorbidities represents the sum of the number of comorbidities that patients possessed including emphysema, diabetes, stroke, and others including 18 different comorbidities in total. Comorbidities are defined in the NACR data as the medical history of conditions which is confirmed with case note review by CR providers by looking at patient’s medical records. A variable was constructed based on whether the patient undergoes the CR prior to the COVID-19 period or during the COVID-19 period and used in the regression model to investigate if COVID-19 is associated with high levels of acute depressive symptoms for CR attenders. Attending CR prior to or during COVID-19 was defined based on the date of the spread of the COVID-19 has been confirmed in the UK which is the end of January. Therefore, 01 February 2019 to 31 January 2020 was defined as pre covid, and the following year after the spread of the disease 01 February 2020 to 31 January 2021 was defined as patients attending during the COVID-19 period. The variables included in the current paper have been chosen in line with the literature and baseline clinical variables that were carried out by CR providers which were explained in detail in previous publications [27–29].

### Statistical analysis

The IBM statistical package for social sciences (SPSS) statistics software V-26 (New York, USA) was used to apply the data analyses. The statistical significance level was set to 5%. Percentages, means, and standard deviations were used to present summary statistics. Using the CR attendance date, a binary variable was constructed, 01.02.2019–31.01.2020 for patients attending CR prior to COVID-19 and 01.02.2020–31.01.2021 for patients attending during the COVID-19 period. Using this variable, baseline characteristics of patients were compared between attending CR during the COVID-19 period and before the COVID-19 period. Baseline characteristics of patients were also compared based on having high and low levels of acute depressive symptoms under the attending CR pre COVID-19 period and during the COVID-19 period. These were using a chi-square test for categorical variables and t-tests for continuous variables. The Cohen’s d effect size was calculated for continuous variables and for categorical variables phi effect size was reported. Finally, in the multivariate analysis, all the variables that have been found to be associated with high levels of acute depressive symptoms at baseline and also based on the previous literature were used adding the attending CR date variable to investigate whether patients attending CR during the COVID-19 period were more likely to have acute depressive symptoms.

## Results

There were 3661 patients with a history of depression who had started CR with valid pre-HADS assessments. 2713 (74.1%) participants with a comorbid history of depression attended CR pre COVID-19 period (01.02.2019–31.01.2020) and 948 (25.9%) participants during the COVID-19 period (01.02.2020–31.01.2021). Patients presented with high levels of acute depressive symptoms pre COVID-19 period was 50.8% (HADS ≥ 8) and this was 48.4% during the COVID-19 period. Figure 1 shows the total population within the study period and the flow diagram shows the different periods and rates of depressive symptoms. In Table 1, the baseline characteristics of patients were compared based on attending CR pre COVID-19 period and during the COVID-19 period. Based on HADS levels, baseline characteristics of patients have been presented in Table 2. Data in these tables include a comparison between the pre COVID-19 period and during the COVID-19 period in these tables.

**Table 1:** Baseline characteristics for pre COVID-19 period and during COVID-19 period groups

Variables	Pre COVID-19 period (n = 2713)		During COVID-19 period (n = 948)		*P*	Effect size
	n	Mean + SD	n	Mean + SD		
Age	2713	61.77 ± 10.98	948	61.73 ± 11.01	0.926	0.00
Total comorbidities	2713	4.84 ± 2.21	948	4.78 ± 2.22	0.514	0.03
Weight	2591	86.60 ± 20.09	876	85.62 ± 19.96	0.212	0.05
HADS anxiety score measurement	2711	9.24 ± 4.79	947	8.79 ± 4.78	0.013	0.09
		%		%		
Gender female %	987	36.3	347	36.8	0.758	0.01
Comorbid anxiety (yes) %	1482	54.6	497	52.4	0.242	0.02
150 min. physical activity a week (yes) %	1019	40.8	339	39.8	0.612	0.01
Smoking (yes) %	377	14.2	130	14.3	0.927	0.00
Partnered %	1445	65.7	478	63.1	0.197	0.02
IMD (most deprived) %	461	21.9	172	22.6	0.698	0.01

*HADS* Hospital Anxiety and Depression Scale, *SD* standard deviation

**Table 2:** Baseline characteristics comparing patients with high and low levels of acute depressive symptoms

Pre COVID-19 period					COVID-19 period			
Variables	HADS < 8 group (n = 1334)	HADS ≥ 8 group (n = 1379)	*P*	Effect size	HADS < 8 group (n = 489)	HADS ≥ 8 group (n = 459)	*P*	Effect size
	Mean (SD)	Mean (SD)			Mean (SD)	Mean (SD)		
Age	63.12 (11.00)	60.47 (10.80)	< 0.001	0.24	63.63 (10.56)	59.70 (11.12)	< 0.001	0.36
Total comorbidities	4.55 (2.13)	5.12 (2.26)	< 0.001	0.26	4.54 (2.16)	5.04 (2.25)	= 0.001	0.23
Weight (kg)	85.25 (18.71)	87.94 (21.30)	= 0.001	0.13	83.95 (19.36)	87.38 (20.45)	= 0.011	0.17
HADS anxiety score measurement	6.48 (3.81)	11.92 (4.06)	< 0.001	1.38	6.18 (3.64)	11.58 (4.25)	< 0.001	1.37
	%	%			%	%		
Female %	36.1	36.4	0.849	0.00	34.2	39.6	0.087	0.05
Comorbid anxiety (yes) %	48.9	60.2	< 0.001	0.11	47.9	57.3	0.004	0.10
150 min. physical activity a week (yes) %	52.5	29.2	< 0.001	0.24	48.7	30.0	< 0.001	0.19
Smoking (Yyes) %	8.4	19.9	< 0.001	0.16	11.3	17.7	0.006	0.09
Partnered %	69.0	62.3	0.001	0.07	67.6	58.3	0.008	0.10
IMD (most deprived) %	15.9	28.1	< 0.001	0.15	16.2	29.8	< 0.001	0.16

Cohen’s d effect size for continuous variables and phi effect size for categorical variables were reported

In Table 1, the baseline characteristics of patients were similar between pre COVID-19 period group and during the COVID-19 period group with no statistically significant difference between most of the characteristics.

Investigating the baseline characteristics participants with high levels of acute post-cardiac event depressive symptoms were younger, have increased weight, higher total number of comorbidities, and had more anxiety compared to patients with low levels of depressive symptoms. And this was similar in both pre COVID-19 period and during the COVID-19 period in Table 2. Moreover, patients with a comorbid history of depression and high levels of acute depressive symptoms at the start of CR were less likely to be physically active, more likely to be smoking, have comorbid anxiety, be single, and be areas of higher social deprivation. This was again similar in pre COVID-19 group and during the COVID-19 period group. Chi-square test findings are also shown in Table 2.

A logistic regression model aimed to test whether attending CR during COVID-19 had an impact on having high levels of acute depressive symptoms and investigate which factors influenced this. The logistic regression model was statistically significant, X^2^(11) = 855.522, *p* < 0.001. The model correctly classified 77.4% of the cases. The model was a good fit based on Hosmer and Lemeshow test (*p* = 0.447). Of the eleven variables, six were statistically significant, HADS anxiety measurement and physical activity, comorbid anxiety, weight, the total number of comorbidities, and IMD (The regression model shown in Table 3).

**Table 3:** Multivariable adjusted odds ratios for having high levels of acute depressive symptoms

Variable	B	SE	Odds ratio	Lower 95% CI	Upper 95% CI
HADS anxiety score measurement	.360	0.018	1.433	1.383	1.484
150 min. a week physical activity (No)	0.780	0.118	2.181	1.730	2.748
Total number of comorbidities	0.102	0.029	1.107	1.045	1.172
IMD (most deprived)	0.292	0.147	1.339	1.004	1.787
Comorbid anxiety	− 0.339	0.129	0.712	0.553	0.917
Weight	0.009	0.003	1.009	1.002	1.015
Year (COVID-19 period)	0.108	0.133	1.114	0.858	1.447

*B* regression coefficient, *SE* standard error, *CI* confidence interval for odds ratio, *IMD* Index of multiple deprivation; the analysis adjusted for age, gender, smoking, and marital status

Although patients attending the CR during the COVID-19 period were 11% more likely to have high levels of acute depressive symptoms, this did not reach statistical significance (OR 1.114, 95%CI 0.858, 1.447). The analysis has also adjusted for age, gender, smoking, and marital status. Increased HADS anxiety score measurement was associated with an increase in the odds of having high levels of acute depressive symptoms compared to low levels of acute depressive symptoms with an odds ratio of 1.433 (95%CI 1.383, 1.484). Being physically inactive had 118% increased odds of having high levels of acute depressive symptoms at the start of CR (OR 2.181, 95%CI 1.730, 2.748). In addition, patients from most deprived areas were 34% more likely to have high levels of acute depressive symptoms (OR 1.339, 95%CI 1.004, 1.787). However, having a comorbid history of anxiety was associated with a reduced likelihood of having high HADS depressive symptom levels.

## Discussion

The current study is the first nationally representative study that investigated the characteristics of patients with a history of depression and compares those characteristics prior to and during the COVID-19 period in UK CR patients. Previous studies explored the impact of COVID-19 on depressive symptoms in the general population often using online questionnaires [15, 16]. However, there is no prior study investigating the impact of COVID-19 on acute depressive symptoms of cardiac patients in the CR setting, particularly in patients with a prior history of depression. The present study also examined the factors associated with acute depressive symptoms at the start of CR and particularly whether attending CR during COVID-19 had an impact on their depressive symptoms. We found that factors that are statistically significantly associated with high levels of acute depressive symptoms in multivariate analysis were physical inactivity, high HADS anxiety symptoms, a higher total number of comorbidities, increased weight, and being from areas of higher social deprivation. Although patients attending CR during COVID-19 were more likely to have high levels of acute depressive symptoms based on HADS measurement, this was not statistically significant.

The prevalence of having higher levels of acute depressive symptoms was similar in pre COVID-19 period at 50.8% (HADS ≥ 8) and during the COVID-19 period (HADS ≥ 8) at 48.4% among patients with a prior history of depression. Although a previous study reported that, in the general population of 1470 individuals from the USA, the prevalence of depressive symptoms was found to be threefold higher than in the preCOVID-19 period during the COVID-19 crisis with 27.8% [16], in the current study the prevalence of high levels of acute depressive symptoms remained similar to the preCOVID-19 period. The reason for this could be that Ettman et al. [16] study only recruited individuals from 31 March 2020 to 13 April 2020 at the start of the COVID-19 pandemic. Whereas our study recruited patients from 01 February 2020 to 31 January 2021 which is a whole year from the commencement of COVID-19 disease in the UK. Therefore, higher levels of depressive symptoms might have been attenuated during a whole year. Furthermore, Ettman et al. [16] applied an online questionnaire for a short period which may lead to selection bias due to only a selection of individuals choosing to participate in their study and they could have different characteristics than the rest of the population. Whereas in the current registry-based study a greater coverage of UK CR patients was recruited over a longer period. Another US-based study recruited 3904 individuals from a general population during a wider period—from June 2020 to January 2021—than the previously stated study using an online questionnaire and found a similar prevalence of high levels of depressive symptoms similar to our study during the COVID-19 period with 52.4%, however, they have used PHQ-9 assessment tool for screening depressive symptoms instead of HADS as in our study [15]. Another reason why the prevalence of high levels of depressive symptoms remained similar pre and during the COVID period in our study could be that patients with a history of depression could have high levels of depressive symptoms based on their specific condition and their depression might be harder to treat compared to patients without the history of depression. Therefore, they may remain with high levels of acute depressive symptoms pre and during COVID-19. Furthermore, patients with a history of depression have a higher mean total number of comorbidities both in pre and during the Covid period (4.84 ± 2.21; 4.78 ± 2.22, respectively) than the general cardiac population, which is 2.67 ± 1.80, during the study period. As the higher total number of comorbidities associated with having higher levels of depressive symptoms, this could be the reason why patients with a history of depression remain with a similar prevalence of acute depressive symptoms both pre and during COVID-19 as their mean total number of comorbidities are also similar in these periods (Table 1).

Another finding was that physical inactivity was influential on having high levels of depressive symptoms at the start of CR. Physically inactive patients were 118% more likely to have high levels of acute depressive symptoms (OR: 2.181, 95%CI 1.730–2.748). A recent systematic review of the general population including 42,293 individuals from 21 studies has found that people performing physical activity on a regular basis and having a stable physical activity routine during the COVID-19 pandemic have shown a lower chance of presenting with depressive symptoms with around 12–32% [30] Our study was supportive of this finding highlighting the importance of keeping moderate physical activity. COVID-19-specific conditions are very likely. Exercising in a group setting, for example, was limited due to social distancing. In addition, due to the closure of sports clubs, gyms, and other common indoor and outdoor areas for physical activity, the COVID-19 pandemic hampered the possibility to be physically active. Although some people were still allowed to walk and jog on the streets, others were not [31]. A lack of opportunities during the pandemic may be linked to less physical activity in general. In addition, the effectiveness of telerehabilitation and home-based CR programmes were also investigated in recent trials [32, 33], these types of programmes may also be useful during the COVID-19 period and help keep the patients that are unable to attend the traditional CR programmes active. However, further research is needed to confirm their effectiveness specifically during the COVID-19 period. A recent study has shown that oral supplementation of L-arginine, a semi-essential amino acid involved in biological processes, enhances the effect of CR on physical performance during the COVID-19 pandemic [34], however, the impact of this supplement on depressive symptoms is not clear. As this supplement is not a usual treatment for CR patients, there is no data available based on this in the audit.

A cross-sectional study by Zhu et al. (2019) included 4043 CVD patients from 16 hospitals in China and participants were enrolled between November 2014 and January 2017 and confirmed the association of physical activity with depressive symptoms. Patient Health Questionnaire-9 (PHQ-9) was used to assess depressive symptoms and PHQ-9 ≥ 10 was accepted as high levels of depressive symptoms. However, previous studies were not inclusive of patients with a prior history of depression attending CR during COVID-19 [35, 36].

In the current study, a higher HADS anxiety score is associated with higher levels of acute depressive symptoms at the start of CR (OR 1.433, 95%CI 1.383, 1.484). A finding which illustrates the interrelationship between anxiety and depression that relate to poor outcomes. A recent Australian retrospective cohort study investigating patients entering CR programmes was in line with this finding [37]. Authors have used Depression Anxiety Stress Scale (DASS-21) measurement tool to assess the patients’ anxiety and depressive symptoms and found that anxiety was strongly associated with depressive symptoms. However, this study was conducted in the pre-covid 19 period and was unable to include patient data from during the COVID-19 period [37]. Weight was another variable associated with high levels of acute depressive symptoms. One kilogramme increase in weight was associated with a 1% increase in the odds of having high levels of depressive symptoms. A previous systematic review conducted on the general population was in agreement with our study [38]. Likewise, weight gain was found to be associated with increased depressive symptoms during COVID-19 in a recent observational study of the general population [39].

One finding is that having a higher total number of comorbidities was associated with 10% increased odds of having high levels of acute depressive symptoms at the start of CR in patients with a prior history of depression (OR 1.107, 95%CI 1.045, 1.172). Another study was contrary to our findings and unable to find an association between comorbidities and depressive symptoms [40]. Yet, the detrimental impact of comorbidities on COVID-19 patients was highlighted in recent studies, and patients with comorbidities are reported to be a vulnerable group who need to be protected from this infectious disease [41]. Our study adds that having a higher total number of comorbidities is also associated with high levels of acute depressive symptoms in CR patients with a prior history of depression. As depression is strongly associated with increased mortality and worse cardiac prognosis for cardiac patients [2, 3], the management of comorbidities may be pivotal and protective not only for deaths related to COVID-19 but also for depressive symptoms in cardiac patients which could be further examined by future studies. However, comorbid anxiety was found to be negatively associated with high levels of depressive symptoms.

The English Index of Multiple Deprivation (IMD) was one of the demographic measurements included in this study and it was found to be associated with high levels of acute depressive symptoms in patients with a prior history of depression after adjusting for age, gender, smoking, marital status, physical inactivity, HADS anxiety, the total number of comorbidities, comorbid anxiety, and attending CR during COVID-19 period. CR patients from most deprived areas were 34% more likely to experience acute depressive symptoms in multivariate analysis. A recent study has found that people living in the most deprived areas have higher depressive symptoms during COVID-19 in the general population [42]. However, this study has some limitations such as recruiting patients only from two weeks of the COVID-19 period in June 2020 and administering a survey by telephone which may have led to the selection of a certain population, only the ones replying to phone calls [42]. Furthermore, the study was not able to factor in the individual’s depressive symptoms prior to COVID-19 as well as whether they had a history of depression which are addressed in our study. In addition, a prior American study has shown that a lower neighbourhood socioeconomic context was associated with a reduced likelihood of starting CR which was measured by the neighbourhood deprivation index [43]. Thus, further strategies need to be developed by CR services to be more inclusive of these socially disadvantaged groups of patients with higher deprivation. Considering the association of deprivation with high levels of depressive symptoms, CR programmes may contemplate screening patients from most deprived areas for depressive symptoms which could be beneficial for early detection of the condition. Indeed, Helmark et al. (2022) study has found that CR patients from areas of higher social deprivation were found to be less likely to be screened for their depressive symptoms. Therefore, the importance of screening for depressive symptoms remains relevant during the COVID-19 period which also aligns with the European Society of Cardiology (ESC) Position statement on psychosocial aspects of cardiac rehabilitation [44].

Lastly, patients with a history of depression attending CR during the COVID-19 period were 11% more likely to have high levels of acute depressive symptoms at the commencement of CR. However, this was not statistically significant adjusting for other covariates (OR 1.114, 95%CI 0.858, 1.447). Although the current literature suggests that in the general population COVID-19 was associated with high levels of depressive symptoms [16, 45], these studies were conducted in a very short period, nearly at the start of the pandemic, and they employed only online questionnaires. However, on the contrary, our study recruited CR patients with a prior history of depression for a whole year during the COVID-19 pandemic. The current study used a nationwide population of UK CR programmes which is representative of the UK CR patients.

### Implications for practice

Patients with a history of depression who are physically inactive, have high anxiety symptoms, increased weight, a higher total number of comorbidities, and from areas of higher social deprivation are more likely to have high levels of acute depressive symptoms. High-risk patients might be more effectively identified if these factors are considered. The current study confirms that assessment of depressive symptoms at the start of CR is important to identify higher-risk patients. Patients with a history of depression need careful monitoring in core CR considering the baseline characteristics of these patients. The recognition, prevention, and treatment of depressive symptoms during COVID-19 remain important as the ongoing situation with restrictions and the spread of new COVID-19 variances across the globe.

### Limitations

The use of an observational approach was a strength of this study to understand the real world by applying routinely collected data. However, our sample size was limited to 948 patients with a history of depression attending CR during the COVID-19 period, larger sample size should be targeted in future studies. Another limitation was our analysis was not able to account for the diagnosis of depression in the CR period, the treatment with antidepressant medication, cardiac function, or the loss of family members due to COVID-19 as this was not recorded in the NACR data set, future research is recommended to take account of these variables. In addition, the influence of the type of the CR programme may also be further studied in the future when comparing post-CR outcomes.

## Conclusions

The aim of the current study was, in patients with prior history of depression, to investigate the association between attending CR during the COVID-19 period and baseline patient characteristics with high levels of acute depressive symptoms when starting CR. Attending CR during the COVID-19 period was associated with a small but non-statistical increase in the odds of having high levels of acute depressive symptoms in patients with a prior comorbid history of depression. As CR programmes return to full capacity our research suggests that the initial CR baseline assessment should also take into account of prior history of depression considering the characteristics of these patients associated with high levels of acute depressive symptoms such as being physically inactive, having increased anxiety, the higher total number of comorbidities and being from most deprived areas. Furthermore, when the patient presents with high levels of acute depressive symptoms, a psychosocial health professional may be consulted, either as part of the multidisciplinary team or as a referral, to ensure proper care and treatment of the patient's symptoms.

## Data Availability

The data that support the findings of this study are available from the National Audit of Cardiac Rehabilitation, but restrictions apply for the availability of this data, which were used under license for the current study, as the data being link anonymised with NHS Digital under section 251 approval, cannot be shared publicly.

## Abbreviations

CR: Cardiac rehabilitation
CVD: Cardiovascular disease
HADS: Hospital Anxiety and Depression Scale
NACR: National Audit of Cardiac Rehabilitation
BHF: British Heart Foundation
MI: Myocardial infarction
PCI: Percutaneous coronary intervention
CABG: Coronary artery bypass graft
IMD: English Index of Multiple Deprivation
